# The Diagnostic Trap in Radiation-Induced Mesothelioma: Kinetic-Morphological Decoupling Masks Molecular Aggression

**DOI:** 10.3390/cancers18020221

**Published:** 2026-01-09

**Authors:** Norikatsu Fujita, Katsumi Fujita, Hironobu Osumi, Yoshiyasu Takefuji

**Affiliations:** 1 Digital Transformation of Skill Unit, The Polytechnic University of Japan, 2-32-1 Ogawa-nishimachi, Kodaira-shi, Tokyo 187-0035, Japan; 2 Department of Thoracic Surgery, Kumamoto University, 1-1-1 Honjo, Chuo-ku, Kumamoto-shi 860-8556, Japanh-osumi@kumamoto-u.ac.jp (H.O.); 3 Faculty of Data Science, Musashino University, 3-3-3 Ariake, Koto-ku, Tokyo 135-8181, Japan; takefuji@keio.jp

**Keywords:** malignant pleural mesothelioma, radiation-induced cancer, kinetic-morphological decoupling, diagnostic trap, CDK4/6 inhibitors, *CDKN2A*, chromothripsis, tumor suppressor genes, precision medicine, therapeutic stratification, CSU Beagle Study

## Abstract

**Background:** In malignant pleural mesothelioma, epithelioid histology is traditionally considered a favorable prognostic marker. However, it remains clinically undetermined whether the intensity of an oncogenic insult can disrupt this link. Radiation-induced cases serve as an unconfounded biological model to dissect such trajectories masked by asbestos confounding. **Methods:** We performed an Individual Patient Data (IPD) synthesis of 20 strictly asbestos-unexposed human cases, applying clinically established dose stratification (intermediate: 20–45 Gy vs. high: >45 Gy). To confirm the observed pattern, we examined data from 829 dogs in the Colorado State University (CSU) Beagle Study. **Results:** In the intermediate-dose group (*n* = 13), a significant positive correlation persisted between age at radiotherapy and the latent period (ρ = 0.567, *p* = 0.043). Conversely, high-dose exposure (>45 Gy) showed a disruption of this age-dependent pattern, with a trend toward inverse correlation (ρ = −0.754, *p* = 0.084). Interaction analysis confirmed a statistically significant divergence between these dose-dependent trends (*p* = 0.005). The CSU Beagle Study (*n* = 829) demonstrated the physical basis of this phenomenon: in the canine model, high-dose exposure (≥0.74 Gy) triggered a “Step-Jump” in cumulative incidence (30.4% at 0.5 years), indicating instantaneous carcinogenic onset distinct from cumulative biological aging. **Conclusions:** This kinetic divergence points to a “Diagnostic Trap.” We propose a ‘Single- to Double-Brake’ framework where intermediate doses preserve age-dependent progression, whereas high doses likely trigger catastrophic genomic failure (chromothripsis) that bypasses the time required for morphological dedifferentiation. Consequently, morphologically indolent epithelioid tumors in high-dose survivors may harbor aggressive molecular profiles not predicted by histology alone, necessitating risk-stratified precision surveillance.

## Simple Summary

Typically, the microscopic appearance of a tumor predicts its biological aggression. However, in malignant pleural mesothelioma caused by radiation, our analysis of 20 rare cases without asbestos exposure suggests that this rule can be clinically deceptive. In this cohort, the intensity of radiotherapy doses appears to shape how the cancer evolves: moderate doses were associated with gradual, age-dependent latent periods, while high doses were associated with rapid, aggressive onset. Paradoxically, these aggressive high-dose tumors retained an indolent-appearing morphology, presenting a potential diagnostic trap that masks their true nature. We propose that reviewing a patient’s radiotherapy history could help expose this discrepancy, potentially guiding risk-stratified precision therapy.

## 1. Introduction

Malignant pleural mesothelioma (MPM) harbors a critical clinical paradox: morphological appearance often fails to predict molecular aggression. While epithelioid histology is traditionally considered favorable as outlined in current ESMO guidelines [1], these tumors can harbor complex, compound deletions of key tumor suppressor genes (TSGs), specifically cyclin-dependent kinase inhibitor 2A (*CDKN2A*) co-occurring with one or more secondary brakes, such as neurofibromatosis type 2 (*NF2*) and/or BRCA1-associated protein 1 (*BAP1*), which confer highly aggressive behavior [2]. This creates what we define as the “Diagnostic Trap”—a clinical scenario where a nominally favorable and indolent epithelioid morphology masks a lethal molecular profile and rapid kinetics. These co-alterations not only modulate therapeutic sensitivity but also potentially lead to resistance to targeted strategies, such as CDK4/6 inhibitors. This trap poses a significant challenge for emerging precision medicine, as evidenced by the heterogeneous outcomes in the Mesothelioma Stratified Therapy (MiST) 2 trial [3], where *CDKN2A* status alone was insufficient to predict treatment response.

To explore these genotype–phenotype disparities, radiation-induced MPM (RI-MPM) serves as an unconfounded biological model, free from the noise of asbestos exposure. Unlike the asbestos-driven pathway where the carcinogenic dose is unquantifiable, RI-MPM correlates a documented radiotherapy dose with the resulting tumor phenotype. Crucially, radiotherapy establishes ‘Event Zero’—a definitive starting point that anchors tumor development to a known physical timeline. This sudden physical impact triggers what Stephens et al. (2011) defined as chromothripsis [4]: a single catastrophic event causing massive genomic rearrangement. We hypothesize that this genomic shattering acts as the biological driver of the ‘Diagnostic Trap’, where the resulting high molecular aggression is decoupled from the tumor’s morphological maturation. Isolating the pure kinetic effects of such a deterministic physical event requires a level of individual clinical granularity that large-scale database studies lack, as they cannot definitively exclude the confounding biological noise of asbestos exposure [5]. Consequently, determining the independent impact of radiotherapy requires prioritizing IPD-level clinical granularity and strict confounding control over statistical scale. By providing a documented temporal starting point, the RI-MPM model uniquely enables the quantitative transformation of the latent period into a surrogate indicator of molecular aggression.

This kinetic–morphological decoupling mirrors findings in other radiation-induced malignancies; for instance, Manner et al. [6] demonstrated that radiation-induced angiosarcomas exhibit distinct molecular signatures, such as high-level MYC amplification, despite remaining morphologically indistinguishable from primary cases. Furthermore, recent clinical data from Lapidot et al. [7] in this Special Issue demonstrate that procedural tract seeding significantly dictates survival in epithelioid MPM. Within our framework, this spatial manifestation of aggressive biology may represent another facet of the ‘Diagnostic Trap’ hidden beneath an indolent morphology.

The aim of this study is to characterize dose-dependent radiation effects in RI-MPM, establishing the latent period as a surrogate indicator of molecular aggression driven by chromosomal disruption, where morphology fails to reflect true tumor behavior. Given the rarity of RI-MPM, our human cohort is inherently limited in size. Therefore, to investigate the physical basis governing this discrepancy, we analyze a large-scale canine dataset (*n* = 829) from the Colorado State University (CSU) Beagle Study to demonstrate how high-dose radiation overrides biological age-dependency.

## 2. Materials and Methods

### 2.1. Data Source and Case Identification

We conducted a systematic compilation to construct a synthesized cohort of asbestos-unexposed RI-MPM cases via an IPD analysis. Cases were identified through a comprehensive literature search of PubMed conducted up to 27 May 2025 (*n* = 173 records), a backward citation search (*n* = 24 records), and a comprehensive review by Sekine et al. [8]. The primary inclusion criterion was a definitive statement of non-exposure to asbestos (occupational, household, or environmental) within the source article. To maintain cohort homogeneity, we also established a strict exclusion criterion for biological evidence of asbestos burden; cases presenting with objective signs (e.g., pleural plaques or asbestos bodies) were excluded even if the exposure history was reportedly negative. Methodological quality of the identified cases was assessed using the Murad et al. framework [9] (Supplemental Table S1).

**Figure 1:**
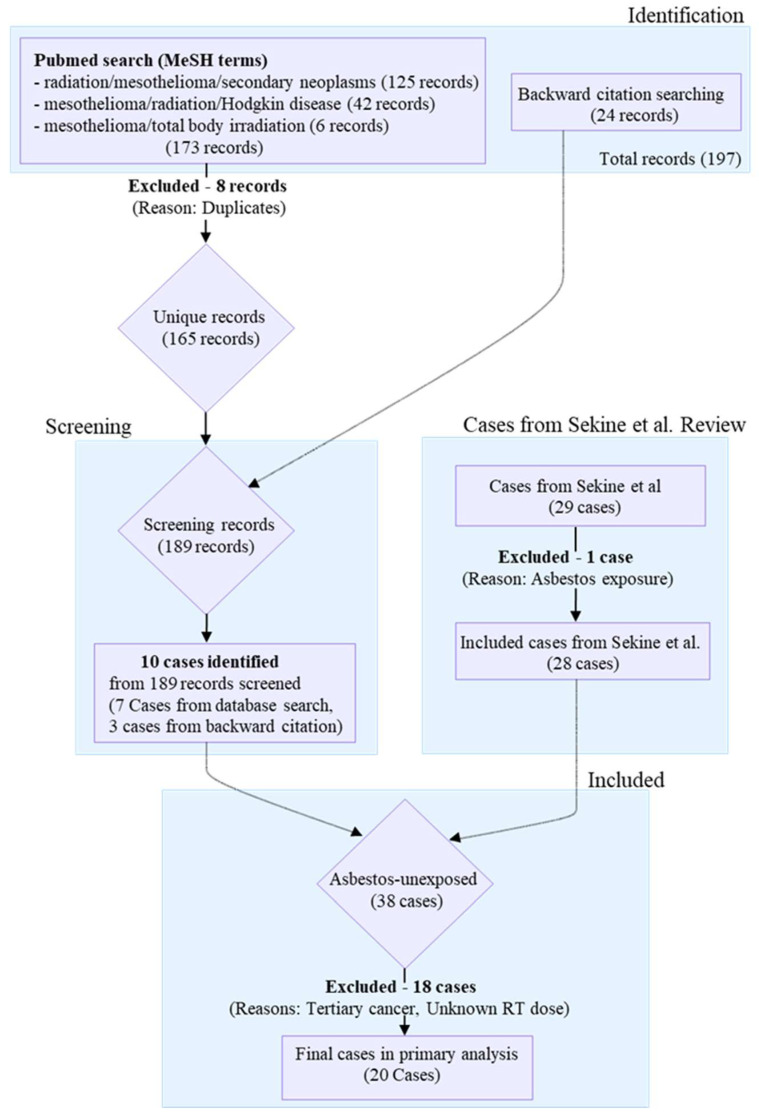
Study Flowchart: Construction of the Asbestos-Unexposed RI-MPM Cohort. The selection process was designed to rigorously exclude confounders for IPD analysis. Cases were identified through PubMed (up to 27 May 2025), backward citation tracking, and prior review. From an initial pool of 197 records, a final cohort of 20 cases was established after excluding 18 candidate cases due to confounding factors (e.g., tertiary cancers, confirmed asbestos exposure) or missing radiotherapy dose data.

The systematic case selection process yielded a candidate cohort of 38 cases (Figure 1). Subsequently, 18 cases were excluded due to confounding factors, including tertiary cancers, confirmed asbestos exposure, missing dose data, or out-of-field development (defined as tumors arising anatomically distant from the primary radiotherapy field or reported explicitly as out-of-field). Consequently, a final cohort of 20 cases was established (Table 1). Notably, these exclusions encompassed the five earliest reports. This incidental exclusion effectively minimized potential bias from historical, less-standardized radiotherapy methods and aggressive combined chemotherapy regimens common in that era [10], thereby refining the cohort for dose-specific analysis.

### 2.2. Molecular Contextualization Using TCGA Data

**Figure 2:**
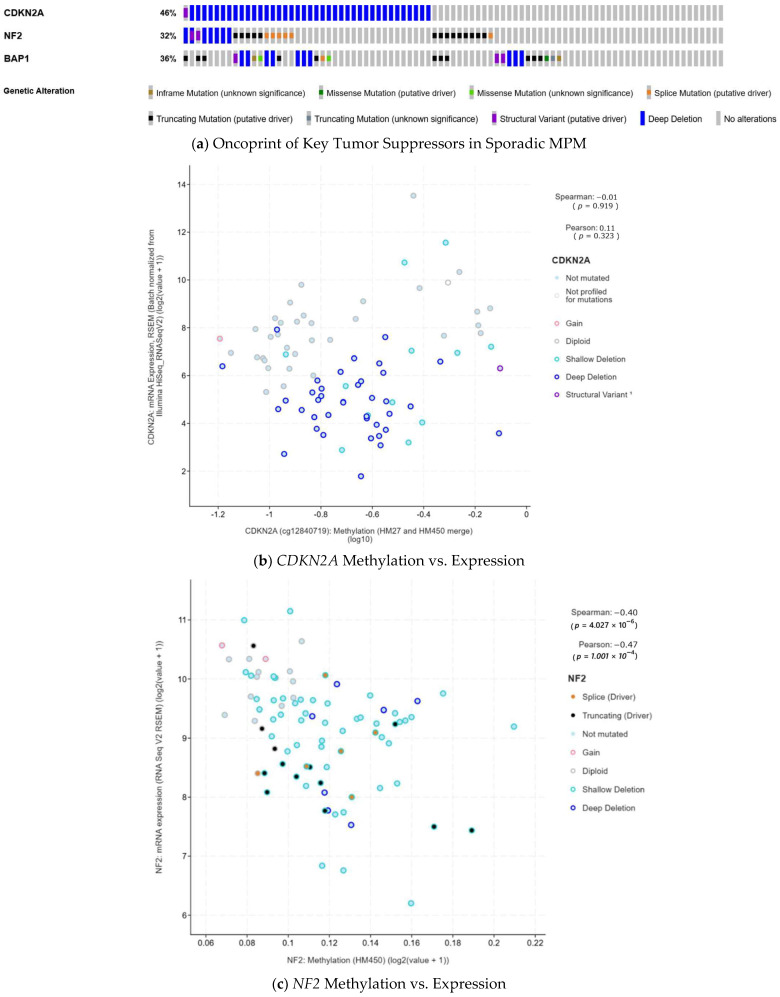
Molecular Landscape of Sporadic Mesothelioma: Distinct Mechanisms of Tumor Suppressor Inactivation. (**a**) Oncoprint of the sporadic Mesothelioma (TCGA, Pan−Cancer Atlas) cohort (*n* = 87) showing frequent co-alterations in *CDKN2A*, *BAP1*, and *NF2*. Mechanisms of inactivation reveal two distinct modes. (**b**,**c**) Mode 1 (Genomic Deletion): Inactivation of *CDKN2A* (**b**) is predominantly driven by deep deletions (*p* = 0.919 for lack of methylation correlation), representing a physical loss of the gene locus. Mode 2 (Epigenetic Suppression): In contrast, *NF2* inactivation (**c**) involves both genomic alterations and epigenetic silencing (*p* < 0.01), supporting the plausibility of regulatory failure via epigenetic mechanisms. This contrast highlights distinct inactivation patterns in sporadic MPM: physical loss (*CDKN2A*) versus combined genomic and epigenetic dysregulation (*NF2*).

To contextualize our clinical findings within the broader molecular landscape of mesothelioma, we analyzed genetic alteration data from the Mesothelioma (TCGA, PanCancer Atlas) cohort (*n* = 87) [2] using the cBioPortal for Cancer Genomics [32,33]. Data were accessed and visualized on August 16, 2025. We examined alteration frequencies and co-occurrence patterns for *CDKN2A*, *NF2*, and *BAP1*, and assessed the correlation between genomic alterations and DNA methylation status using cBioPortal’s standard statistical tools and visualization functions (Figure 2).

### 2.3. Rationale for Dose Stratification

The stratification thresholds (20 Gy and 45 Gy) were adopted directly from NCCN guidelines [34] and clinical evidence from radiation-induced sarcoma studies [35] to reflect distinct treatment intents (palliative vs. curative). Crucially, these external criteria were applied to ensure categorization remained independent of our dataset distribution:

First, regarding the low-dose threshold (<20 Gy), while doses in this range are insufficient for definitive local control of adult solid tumors, they are clinically critical for palliative care or curative pediatric protocols, such as Whole Lung Irradiation (WLI) for Wilms’ tumor and Total Body Irradiation (TBI) for leukemia. Radiobiologically, while sufficient to induce mutations, these doses are generally insufficient to trigger the catastrophic genomic shattering characteristic of high-dose sequelae.

Second, the intermediate-dose threshold (20–45 Gy) corresponds to standard curative protocols for hematologic malignancies. According to NCCN Guidelines, involved-site radiation therapy (ISRT) for Hodgkin lymphoma typically ranges from 20 to 30 Gy for non-bulky disease to 30–36 Gy for bulky disease, with escalation up to 36–45 Gy for refractory cases. Consequently, this window represents a “moderate-intensity” therapeutic zone: sufficient to induce significant DNA damage and cell death in radiosensitive tissues but generally remaining below the intensity required for definitive control of radioresistant solid tumors.

Finally, the high-dose threshold (>45 Gy) represents intensive regimens required for solid tumor control, such as definitive radiotherapy for breast cancer or sarcomas. At these levels, the biophysical impact on the genome intensifies, significantly increasing the probability of complex chromosomal rearrangements and compound genetic losses. Clinically, this threshold is corroborated by large cohort studies of radiation-induced sarcoma, where the vast majority of cases occurred after doses exceeding 45 Gy [35].

### 2.4. Statistical Analysis

We analyzed the associations among age at radiotherapy, latent period, and radiotherapy dose using a multi-step approach.

#### 2.4.1. Assessment of Non-Linearity and Justification for Stratified Analysis

Prior to formal statistical analysis, we assessed the global relationship between age at radiotherapy and the latent period across the entire synthesized cohort (*n* = 38). Visual inspection revealed a complex, non-linear distribution (Supplemental Figure S1), indicating that a single linear regression model would be statistically inappropriate for the pooled dataset. To resolve this biological heterogeneity and investigate whether therapeutic intensity drives these divergent patterns, we adopted a stratified analysis model applying the clinically established dose thresholds (low, intermediate, and high) defined in Section 2.3.

#### 2.4.2. Regression and Correlation Analysis

Following radiotherapy dose stratification, we operated under the assumption that linearity is preserved within each dose-specific subgroup, unlike the non-linear aggregate data. For the statistical modeling, we designated the intermediate-dose group (*n* = 13) as the subject of our primary analysis, as it represents the largest and most clinically homogeneous subgroup. We modeled the kinetic relationship using linear regression and quantified the correlation strength using Spearman’s rank-order correlation coefficient. Simultaneously, we performed an exploratory analysis on the high-dose group (*n* = 6). Although sample size limitations precluded definitive independent conclusions, regression lines were fitted to this group specifically to enable a comparative interaction analysis against the primary intermediate-dose baseline. The single low-dose case (*n* = 1) was excluded from regression modeling.

#### 2.4.3. Assessment of Model Stability and Sensitivity Analysis

We assessed the internal stability of the regression model using a leave-one-out approach. To specifically verify the robustness of this association against influential outliers, we also performed a sensitivity analysis by excluding the youngest (Case 6, 6 years) and oldest (Case 21, 40 years) patients in the intermediate-dose group. To evaluate the robustness of the associations in each dose group, we generated 95% confidence intervals (CI) for Spearman’s rank correlation coefficient via bootstrap analysis (10,000 iterations) to determine whether the intervals included zero. Additionally, we qualitatively assessed chemotherapy’s influence by examining whether cases receiving specific regimens deviated from the 95% CI of the regression line.

#### 2.4.4. Interaction Analysis: Comparison of Dose Groups

To statistically validate the divergence observed between the primary (intermediate-dose) and exploratory (high-dose) trends, we employed an Analysis of Covariance (ANCOVA). Specifically, we tested the interaction term (Dose Group × Age at Radiotherapy) to determine if the regression slopes were significantly distinct. Prior to interpretation, we verified model assumptions: Levene’s test confirmed homogeneity of variance (*F* = 1.14, *p* = 0.30), and the Shapiro–Wilk test confirmed normality of residuals (*W* = 0.947, *p* = 0.355). No influential outliers were detected (maximum Cook’s distance = 0.142, well below the standard threshold of 1.0). All statistical analyses were performed using R statistical software (version 4.5.1, R Foundation for Statistical Computing, Vienna, Austria). Statistical significance was defined as *p* < 0.05. The R code used for these regression and correlation analyses is provided as File S1 in the Supplementary Materials.

### 2.5. Quantitative Validation of “Event Zero” Dynamics via Cumulative Incidence Analysis

To validate the “Event Zero” theory and distinguish immediate physical disruption from gradual biological aging, we utilized the CSU Beagle Study dataset from the Northwestern University Radiation Archive (NURA) [36]. This dataset originates from a large-scale, lifelong study of beagles exposed to ionizing radiation at various ages, as documented in the 1989 Annual Report [37]. Purebred Beagles were selected to minimize “biological noise” and isolate the fundamental kinetic laws governing carcinogenesis.

From the initial repository of 30,070 individuals, including both irradiated and control animals, we extracted cases with terminal disease requiring euthanasia (TERMCODE 42), representing various radiation-induced cancers. This heterogeneity is appropriate for testing the Event Zero hypothesis, as chromothripsis-driven carcinogenesis operates at the chromosomal level independently of specific cancer types. Cases irradiated in utero (age at exposure < 0) and those that died within 0.1 year of irradiation (latent period < 36.5 days) were excluded to focus on postnatal exposure effects. This resulted in a total analysis population of *n* = 829 classified into three dose groups based on the experimental design: 0 Gy (control, *n* = 144), ~0.16 Gy (range 0.151–0.1775 Gy, *n* = 175), and ≥0.74 Gy (range 0.7457–5.2812 Gy, *n* = 510).

To characterize the temporal dynamics of tumor onset, we performed a Cumulative Incidence Analysis. Instead of relying on linear correlation models, we calculated the cumulative event rate at discrete time points (0.5, 1.0, 2.0, 3.0, 5.0, 7.0, 10.0, 12.0, 15.0, and 20.0 years) across the three dose groups. This approach was adopted to detect “Step-Jump” kinetics—specifically, a disproportionately high baseline incidence in the ultra-early phase (e.g., at 0.5 years). Such a pattern serves as a distinct signature of “Event Zero,” reflecting a catastrophic physical event that triggers immediate tumor initiation, in contrast to the gradual, time-dependent accumulation characteristic of biological aging. The R code specifically developed for this cumulative incidence analysis is provided as File S2 in the Supplementary Materials.

## 3. Results

### 3.1. Human RI-MPM Cohort Characteristics and Dose-Stratified Trends

**Figure 3:**
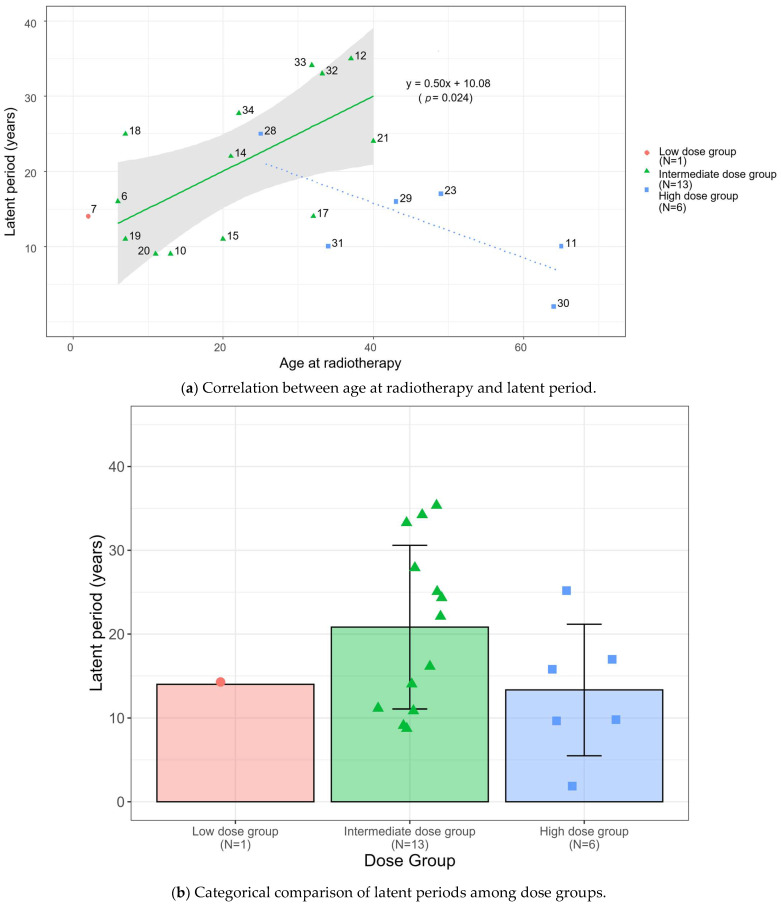
Dose-Dependent Divergence in the Relationship Between Age at Radiotherapy and Latent Period. (**a**) Scatter plot illustrating the positive correlation in the intermediate-dose group (solid green line; *y* = 0.50*x* + 10.08, *p* = 0.024) and the divergent trend in the high-dose group (dotted blue line). Each point represents an individual case, with shapes indicating dose categories: low (pink circle), intermediate (green triangle), and high (blue square). The gray shaded area represents the 95% confidence interval for the intermediate-dose group. (**b**) Bar diagram showing the mean latent period for low- (*n* = 1), intermediate- (*n* = 13; mean latent period 20.8 ± 9.9 years), and high-dose (*n* = 6; mean latent period 13.3 ± 7.9 years) groups. Individual data points are overlaid to show the distribution within each cohort, with shapes and colors consistent with panel (**a**). Error bars represent the standard deviation (SD), illustrating the biological variability in latent period relative to the radiation dose.

The observation of a complex, non-linear pattern between age at radiotherapy and latent period (Supplementary Figure S1) prompted a dose-stratified analysis to clarify the underlying trends. As detailed in the Methods (Section 2.3), cases were stratified into three groups (low: <20 Gy; intermediate: 20–45 Gy; and high: >45 Gy) applying external clinically established thresholds independent of the dataset distribution. This stratification uncovered opposing trends between the main radiotherapy dose groups: a significant positive correlation in the intermediate-dose group and a non-significant negative trend in the high-dose group (Figure 3).

Histological subtypes were reported in 14 of the 20 cases in the final cohort (Table 1). Epithelioid histology was the dominant subtype across all dose groups. In the intermediate-dose group (*n* = 13), histology was documented in 10 cases, of which 8 (80%) were epithelioid and 2 (20%) were biphasic. In the high-dose group (*n* = 6), histology was documented in 3 cases, all of which were epithelioid. Notably, the two biphasic cases (Case 14 and Case 32) occurred at the higher end of the intermediate-dose range (40 Gy) in young patients (ages 21 and 33). Crucially, Case 14 developed a biphasic tumor despite receiving radiotherapy alone, while Case 32 received concurrent chemotherapy. In contrast, despite higher radiotherapy intensity, no biphasic or sarcomatoid cases were documented in the high-dose group among reported cases.

This scatter plot reveals statistically significant divergent trends between the main dose groups. The intermediate-dose group (*n* = 13) shows a significant positive correlation (*R*^2^ = 0.385, *p* = 0.024; Spearman’s *ρ* = 0.567, *p* = 0.043). In contrast, the high-dose group (*n* = 6) shows a non-significant negative trend (Spearman’s *ρ* = −0.754, *p* = 0.084). Crucially, an exploratory Analysis of Covariance (ANCOVA) indicated that the difference between these opposing regression slopes is significant (*p* = 0.005 for interaction). Numbers correspond to Case IDs in Table 1. Furthermore, categorical comparison of the mean latent periods (Figure 3b) revealed that the intermediate-dose group exhibited a longer average latent period (20.8 ± 9.9 years) compared to the high-dose group (13.3 ± 7.9 years), although the distribution within each cohort showed biological variability.

### 3.2. The Intermediate-Dose Group: A Significant Positive Correlation

In the intermediate-dose group (*n* = 13), we observed a significant positive correlation between age at radiotherapy and the latent period (*R*^2^ = 0.385, *p* = 0.024; Spearman’s *ρ* = 0.567, *p* = 0.043).

Leave-one-out cross-validation, using the *p*-value derived from the linear regression model, revealed sensitivity to influential cases, with the correlation remaining statistically significant (*p* < 0.05) in 11 of 13 iterations (*p*-range: 0.008–0.066). Bootstrap analysis (10,000 iterations) yielded a 95% CI of [0.068, 0.883] (Supplemental Figure S2A), which does not include zero.

To verify that this association was not driven by leverage points, we performed a sensitivity analysis by excluding the youngest (Case 6, 6 years) and oldest (Case 21, 40 years) patients. The correlation remained statistically significant after excluding these extremes (Spearman’s *ρ* = 0.693, *p* < 0.05), suggesting that the age-dependent pattern is consistent across the intermediate-dose cohort.

### 3.3. The High-Dose Group: An Opposing Negative Trend

In contrast to the intermediate-dose group, the high-dose group (*n* = 6) displayed a reversal of the age-latency correlation (Spearman’s *ρ* = −0.754, *p* = 0.084). While this trend did not reach statistical significance due to the limited sample size, the directional shift is consistent with the kinetic pattern in the high-dose group of the CSU Beagle Study (≥0.74 Gy; *n* = 829 total; see Section 3.5), where biological age effects are effectively neutralized (near-zero slope) despite statistical correlation.

### 3.4. Exploratory Comparison: Interaction Between Radiotherapy Dose and Age at Radiotherapy

Exploratory ANCOVA indicated a significant interaction between radiotherapy dose group and age at radiotherapy (*F* (1,15) = 10.58, *p* = 0.005, *ηp*^2^ = 0.41), indicating that the regression slopes for the intermediate- and high-dose groups are statistically distinct. To distinguish this rapid onset from a statistical artifact of shorter life expectancy in older patients (competing mortality), we utilized the intermediate-dose group as a biological baseline. This group demonstrates that latent period naturally extends with increasing age at radiotherapy. The fact that the high-dose group deviates from this baseline—exhibiting uniformly short latent period regardless of age—strongly suggests that the phenomenon represents genuine biological acceleration rather than survival truncation.

### 3.5. Temporal Signatures Distinguish Instantaneous from Cumulative Carcinogenesis

The temporal pattern of terminal disease onset fundamentally differs between dose groups, suggesting distinct carcinogenic mechanisms. The cumulative incidence analysis (Table 2; visualized in Figure 6) demonstrates a striking divergence in early onset rates.

At just 0.5 years post-exposure (excluding deaths within 0.1 years), the ≥0.74 Gy group exhibited a 30.4% cumulative incidence, compared to 1.4% in controls. This 22-fold elevation occurred within six months—a timeframe biologically insufficient for the sequential mutation accumulation typical of multi-stage carcinogenesis. As shown in Figure 6, two qualitatively distinct patterns. The control group follows a gradual, time-dependent linear increase, consistent with the stochastic accumulation of mutations due to biological aging. In stark contrast, the ≥0.74 Gy group exhibits a “Step-Jump” pattern: an immediate, vertical rise in incidence at the initial time point, followed by a parallel progression. This “zero-time” elevation suggests that a substantial proportion of tumors originated from instantaneous mutagenic events at the moment of irradiation (“Event Zero”), effectively bypassing the latent period required for gradual biological evolution. This phenomenon appears dose-threshold-dependent. The 0.16 Gy group showed the slowest kinetics (0.5-year incidence: 1.7%). However, this paradoxically low rate likely reflects a selection bias rather than a protective effect; this group exhibited a higher rate of early exclusions (deaths < 0.1 years post-exposure). This suggests the early removal of highly susceptible individuals, leaving a more robust cohort. Thus, only doses exceeding a physical threshold appear sufficient to trigger the instantaneous “Event Zero” mechanism. Notably, the large initial difference between high-dose and control groups progressively diminished as control group cases accumulated over time, consistent with radiation accelerating the kinetics of onset by bypassing the stochastic mutational process, rather than increasing the ultimate lifetime risk of cancer development.

## 4. Discussion

### 4.1. Proposal of the ‘Single- to Double-Brake Failure’ Framework

Our IPD analysis of definitively asbestos-unexposed RI-MPM cases (*n* = 20) provides the clinical basis for a unified physical framework, revealing a statistically significant dose-dependent divergence in clinical presentation (*p* = 0.005, *ηp*^2^ = 0.41). Specifically, intermediate-dose radiotherapy (20–45 Gy) showed a positive correlation between age at radiotherapy and latent period, whereas high-dose exhibited a pattern consistent with instantaneous onset (“Event Zero”), as quantitatively validated in the CSU canine model. This pattern challenges the prevailing assumption that epithelioid mesothelioma represents a biologically uniform entity and suggests distinct biological mechanisms underlying radiation-induced carcinogenesis at different dose levels. The molecular landscape of sporadic mesothelioma, predominantly asbestos-driven, identifies *CDKN2A*, *NF2*, and *BAP1* as dominant tumor suppressors (Figure 2a). While the etiology differs from RI-MPM, these genes provide an established framework for hypothesis generation. Their inactivation mechanisms vary: *CDKN2A* predominantly undergoes physical deletion (Genomic Deletion), while *NF2* often involves epigenetic silencing (epigenetic suppression) (Figure 2b,c). We leverage this distinction—not as direct evidence for RI-MPM—but as a mechanistic scaffold to interpret our dose-stratified clinical observations.

**Figure 4:**
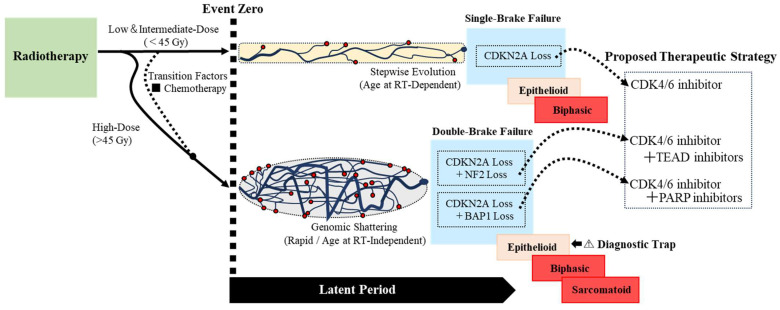
Proposed ‘Single- to Double-Brake Failure’ Concept for Dose- Dependent Pathogenesis in RI-MPM. This conceptual framework integrates clinical latent period trends with tumor suppressor biology. Top Path (Single-Brake): We postulate that intermediate-dose radiotherapy induces stepwise evolution (e.g., via small-scale mutations or functional silencing), while secondary brakes (*NF2*) presumably remain functional. This gradual pathway preserves morphological plasticity (potential for EMT), resulting in age-dependent latent period. Bottom Path (Double-Brake): High-dose radiotherapy likely precipitates a catastrophic physical genomic deletion of the Master Clock and multiple secondary safety brakes (including *NF2* and/or *BAP1*) potentially via mechanisms such as chromothripsis. This rapid expansion bypasses morphological adaptation (Type 3 EMT), resulting in “Decoupled” Epithelioid tumors—a Diagnostic Trap where an indolent appearance masks aggressive compound resistance. Blue Circles: Surviving tumor-initiating clones (size ∝ aggressiveness). Red Circles: Irradiated cells eliminated via senescence or apoptosis (Cell Killing Effect). Note: This diagram is a simplified conceptual schematic intended to visualize the proposed ‘Single- to Double-Brake’ hypothesis based on the observed trends in the clinical latent period. It does not exhaustively depict the complex molecular signaling cascades or all potential interaction partners of *NF2* and/or *BAP1*. The model serves as a heuristic framework to guide future molecular validation studies.

Synthesizing the molecular scaffold with our clinical data, we propose the ‘Single- to Double-Brake Failure’ model as a mechanistic hypothesis consistent with established chromothripsis biology (Figure 4). In this hierarchical system, we designate *CDKN2A* as the ‘Master Clock’ regulating cellular senescence, analogous to its role in the epigenetic aging process [38]. Crucially, radiotherapy acts as “Event Zero,” establishing a synchronized start point for carcinogenesis. This suggests that the kinetic trajectory is likely anchored to age at radiotherapy, but this dependency is overridden by the intensity of the genotoxic insult (high-dose radiotherapy or concurrent chemotherapy). In our proposed framework, ‘Single-Brake’ and ‘Double-Brake’ states can be conceptually distinguished by these latency kinetics.

Single-Brake Failure (Stepwise Evolution): This state, characteristic of the intermediate-dose group, represents the isolated disruption of the Master Clock. We postulate that intermediate-dose radiotherapy is a “moderate-intensity” insult—sufficient to functionally compromise *CDKN2A* via small-scale mutations or methylation but lacking the destructive force to cause widespread genomic shattering. Consequently, the gene locus is likely structurally retained but functionally silenced, representing a “soft” failure while secondary regulators like *NF2* remain intact. Because the gene locus is physically retained, the carcinogenic process follows a gradual trajectory. This explains the linear correlation we observed: the “Single-Brake” failure allows tumorigenesis to proceed, but the speed of progression remains tethered to the host’s biological age. This correlation can be mechanistically interpreted through the lens of proliferative kinetics. Although younger tissues typically possess robust DNA repair mechanisms, their high baseline proliferative activity functions as a kinetic accelerator. Consequently, rapid cell turnover propagates the initial ‘Single-Brake’ dysregulation more efficiently than in the quiescent tissues of older individuals, shortening the interval required to reach the malignant threshold despite their higher repair potential.

Double-Brake Failure (Genomic Shattering): In stark contrast, ‘Double-Brake Failure’ represents a catastrophic compound loss of the Master Clock and one or more secondary safety brakes (e.g., *CDKN2A* in combination with *NF2* and/or *BAP1*), predominantly driven by high-dose radiotherapy. We propose that this high-intensity insult triggers genomic shattering (e.g., chromothripsis), leading to the physical genomic deletion of both the Master Clock and secondary Safety Brakes. This aligns with the concept of genomic instability as an enabling characteristic of cancer [39], effectively bypassing the senescence barrier. Consequently, the tumor likely acquires aggressive growth properties instantaneously at “Event Zero,” resulting in a rapid onset that bypasses the dependency on age at radiotherapy. The biological necessity of this ‘bypass’ mechanism is strikingly illustrated by the counter-factual analysis of Case 30 (66 years old, 60 Gy). Based on the biological baseline established by the intermediate-dose group (*y* = 0.50*x* + 10.08), the theoretical latent period for a 66-year-old would be approximately 43.08 years, placing the projected onset at age 109. Biologically, this implies that under ‘Single-Brake’ kinetics, the tumor would never manifest clinically due to censorship by natural death. However, the patient developed the disease in just 2 years. The fact that the high-dose insult forced a tumor—which should have been masked by competing mortality—to manifest immediately suggests a fundamental biological acceleration. This supports the hypothesis that high-dose mechanisms may functionally dismantle the Master Clock entirely, overriding not only the age-dependency but also the protective constraints of time itself.

### 4.2. Potential Role of Chemotherapy as a Kinetic Modifier

**Figure 5:**
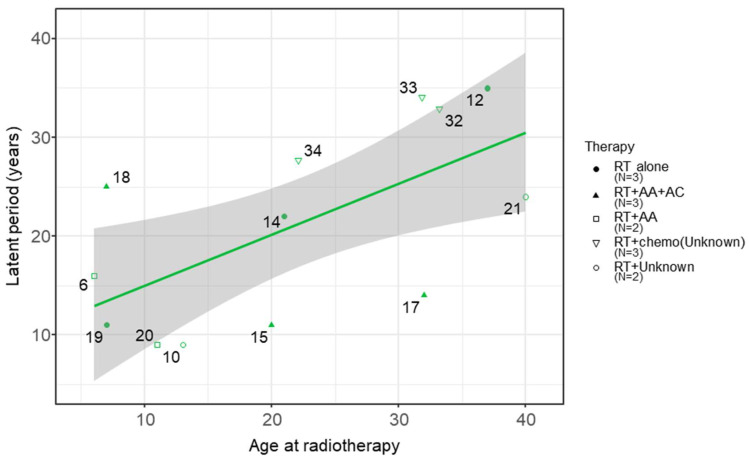
Prior Chemotherapy as a Potential Modifier of the Relationship between Age at Radiotherapy and Latent Period Relationship in the Intermediate-Dose Group. The green solid line and shaded area represent the regression line and its 95% confidence interval, respectively. This scatter plot visualizes cases from the intermediate-dose group, stratified by treatment history. Cases treated with radiotherapy alone (RT alone; solid circles) consistently fall within the 95% confidence interval (CI) of the main regression line. In contrast, cases with prior chemotherapy show greater deviation. While several cases receiving DNA-damaging agents (e.g., alkylating agents) fell below the 95% CI, indicating potential acceleration, the overall pattern reflects increased heterogeneity. This suggests that prior chemotherapy functions as a compounding genotoxic factor that disrupts the predictable relationship between age at radiotherapy and latent period observed in RI-MPM.

While radiotherapy dose appears to primarily shape latency kinetics, our data suggest that concurrent chemotherapy may act as a critical kinetic modifier. Our analysis revealed that patients receiving DNA-damaging agents within the intermediate-dose group exhibited pronounced heterogeneity. While specific cases (e.g., Case 10, 15 and 17) demonstrated accelerated onset indistinguishable from the high-dose phenotype, others (e.g., Case 18, 33 and 34) retained extended latent periods consistent with the radiotherapy-only trajectory (Figure 5).

We propose that this distinct heterogeneity may clinically reflect stochastic compound genotoxicity. Unlike high-dose radiotherapy, which likely obliterates genomic barriers with high efficacy, systemic chemotherapy induces stochastic physical damage to the genome. We postulate that chemotherapy accelerates tumorigenesis only when it physically disrupts a secondary “Safety Brake” (e.g., *NF2*) in a clone already compromised by radiotherapy. Consequently, this “Second Hit” functions as a probabilistic “Double-Brake” converter, uncoupling the relationship between age at radiotherapy and latent period in a subset of patients while sparing others, thereby driving the observed clinical scatter.

### 4.3. Mechanistic Interpretation: Potential Divergence in Evolutionary Trajectories

The central paradox in our cohort is the dissociation between morphology and clinical behavior in high-dose cases (>45 Gy), which exhibit aggressive onset yet retain epithelioid histology. To interpret this dissociation, it is necessary to distinguish the specific biological context of the Epithelial–Mesenchymal Transition (EMT). EMT is classified into three functional subtypes: Type 1 (embryogenesis), Type 2 (fibrosis), and Type 3 (carcinoma progression). In the standard pathogenesis of mesothelioma, Type 3 EMT is the established mechanism for acquiring invasiveness, driven by transcriptional reprogramming that necessitates a morphological shift from epithelioid to sarcomatoid histology.

The absence of this morphological shift is consistent with the hypothesis that tumorigenesis in this context may bypass the standard Type 3 EMT program. We interpret this divergence through the lens of evolutionary concepts of “Gradualism” versus “Punctuated Equilibrium” described by Stephens et al. [4]. Although they proposed ionizing radiation as a plausible driver of chromothripsis, our findings offer rare clinical insight consistent with this catastrophic mechanism, suggesting that therapeutic intensity can compress evolutionary time. Intermediate-dose radiotherapy appears to mirror the “gradualism” model, where mutations likely accumulate sequentially over decades. This gradual timeline provides the biological window necessary for phenotypic evolution via the Type 3 EMT program. Unlike the high-dose group, this trajectory preserves morphological plasticity, as evidenced by the presence of biphasic cases within this cohort. This suggests that the gradual ‘Single-Brake’ progression provides sufficient time for partial EMT, allowing evolution into a biphasic phenotype, whereas the majority remain epithelioid. Crucially, Case 14 (40 Gy, RT-alone) exhibited biphasic histology yet demonstrated age-dependent kinetics, aligning it with the Single-Brake framework. This specific case illustrates that, for intermediate-dose insults, morphological progression (EMT) may be primarily a function of time (gradualism), not an immediate sign of compound genetic failure. In contrast, high-dose radiotherapy has been shown to trigger chromothripsis in experimental models and certain solid tumors—a catastrophic event involving massive genomic rearrangement. We postulate that this mechanism acts as an evolutionary “shortcut.” If cells acquire malignant potential (e.g., via simultaneous physical deletion of *CDKN2A* and *NF2*) instantaneously, they effectively bypass the gradual signaling cascades required for Type 3 EMT. Consequently, the tumor acquires invasiveness directly through genomic disruption rather than morphological adaptation. The biological distinctiveness of this pathway is effectively illustrated by Case 28 (50 Gy); even with a prolonged 25-year latent period—normally sufficient for EMT—the phenotype remained ‘locked’ in an epithelioid state. This implies that genomic damage compromised the capacity for morphological drift.

The rare historical cases (Cases 1, 2) that exhibited rapid onset with sarcomatoid histology do not contradict this model but reflect the specific therapeutic context of the 1960s and 1970s [10]. As confirmed in the original reports by Brody et al., these cohorts were treated with aggressive combined-modality protocols. We speculate that the sarcomatoid conversion in these cases may be attributed to compound therapeutic toxicity: while radiotherapy provided the kinetic acceleration, the extreme systemic stress from alkylating agents likely forced the activation of stress-responsive Type 3 EMT pathways, superimposing morphological dedifferentiation onto the kinetically aggressive clone.

### 4.4. Therapeutic Implications: The Diagnostic Trap and Risk Stratification

This kinetic-morphological decoupling creates a potential “diagnostic trap.” This challenge is compounded by the inherent difficulty of mesothelioma diagnosis; indeed, despite recent improvements, diagnostic error rates remain substantial, ranging from approximately 14% in high-resource settings to 50% in developing regions [40]. In the high-dose context (>45 Gy), an epithelioid tumor effectively masquerades as a simple “Single-Brake” case while potentially harboring aggressive “Double-Brake” compound resistance. This dose-dependent model offers a mechanistic explanation for the therapeutic heterogeneity observed in the sporadic setting, such as the MiST2 trial [3]. In that trial, 54% of patients failed to respond despite having *CDKN2A*-deficient tumors. We postulate that these non-responders biologically mirror our high-dose “Double-Brake” phenotype. While CDK4/6 inhibitors effectively blockade the cell cycle downstream of *CDKN2A* loss, the concurrent loss of a secondary safety brake (e.g., *NF2* or *BAP1*)—whether caused by high-dose radiotherapy in RI-MPM or stochastic accumulation in sporadic MPM— potentially activates alternative oncogenic signaling (e.g., Hippo/YAP pathway). This likely establishes a bypass mechanism that sustains cell proliferation despite CDK4/6 inhibition. Based on this biological parallelism, we propose a risk-stratified strategy for RI-MPM.

Low-to-Intermediate-Dose Radiotherapy (≤45 Gy) [RT-Alone]: Epithelioid tumors in this range likely reflect a “Single-Brake” state where the secondary brake (*NF2*) remains intact, as the radiotherapy intensity was insufficient to cause compound genomic shattering. Thus, dependency on the CDK4/6-RB pathway is likely preserved, making them potential candidates for CDK4/6 inhibitor monotherapy [39]. This biological consistency is corroborated by a recent report of epithelioid RI-MPM following low-dose Total Body Irradiation (12 Gy), which exhibited a characteristic long latent period consistent with Single-Brake biology [41].

High-Dose Radiotherapy (>45 Gy) and Chemotherapy-Associated Cases: Despite their benign epithelioid appearance—a result of rapid growth outpacing morphological dedifferentiation—these tumors carry a high risk of compound deficiencies. Since bypass mechanisms are likely active, CDK4/6 inhibitors alone are predicted to be insufficient. Molecular assessment of *NF2* and/or *BAP1* status appears warranted, particularly for high-dose cases. While immunohistochemistry (IHC) for *BAP1* and MTAP (a surrogate for *CDKN2A*) serves as an accessible first-line screen in clinical practice [1], comprehensive genomic profiling (e.g., NGS) is particularly warranted for high-dose cases to capture complex co-alterations like *NF2* loss [42]. Detecting potential compound resistance is crucial to justify redirecting patients from ineffective CDK4/6 inhibitor monotherapy toward synthetic lethality approaches (e.g., TEAD inhibitors) targeting these specific pathways [43].

### 4.5. From Chromothripsis to Clinical Phenotype: Cross-Species Analysis

**Figure 6:**
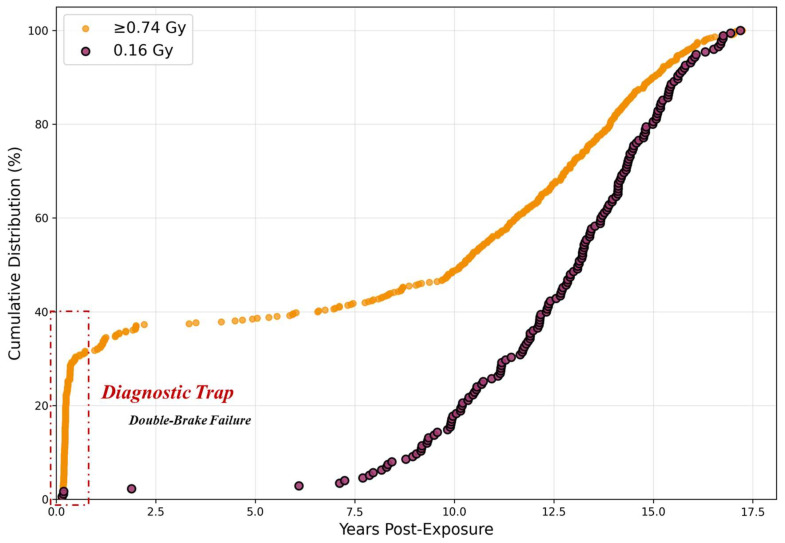
Cumulative Incidence of Radiation-Induced Terminal Disease in the CSU Beagle Study (Visualizing Table 2). The graph visualizes the cumulative incidence data from Table 2. Purple Line (0.16 Gy): Represents the 0.16 Gy group. The curve shows a gradual, linear increase over time, consistent with standard biological progression. Orange Line (≥0.74 Gy): Represents the ≥0.74 Gy group. Note the distinct “Step-Jump” at the origin, where incidence spikes to 30.4% almost immediately (within 0.5 years). This vertical shift indicates that a subset of tumors was induced instantaneously by the physical impact of radiation (“Event Zero”), independent of biological aging time.

The “Step-Jump” kinetics identified in the CSU Beagle Study (Figure 6) provide strong cross-species validation for a distinct carcinogenic mechanism at high doses. While the control and 0.16 Gy groups exhibited a gradual, time-dependent increase consistent with Darwinian gradualism, the ≥0.74 Gy group demonstrated an immediate vertical rise. This “zero-time” onset implies that the carcinogenic timeline was compressed into a single instant—an “Event Zero”—rather than spread out over years of biological aging. We propose that this kinetic signature is the macroscopic manifestation of chromothripsis. The dose-dependent patterns observed here align perfectly with the three defining characteristics of chromothripsis established by Stephens et al. [4], offering a mechanistic explanation for the “Diagnostic Trap. “First, regarding massive genomic rearrangement: Stephens et al. define chromothripsis as a phenomenon distinct from gradual mutation accumulation, involving widespread genomic destruction. High-dose radiation acts as the physical driver for this event. The energy deposited by high-dose irradiation generates clustered DNA double-strand breaks that exceed cellular repair capacity, triggering catastrophic chromosomal fragmentation and random reassembly. The near-zero slope observed in the high-dose group (Figure 6) reflects this non-cumulative, instantaneous physical shattering. Second, concerning the inference of a “one-off cellular crisis”: The critical conclusion drawn by Stephens et al. is that these rearrangements occur in a single event, rejecting the model of sequential acquisition. Our kinetic analysis supports this “single crisis” model. If tumorigenesis were driven by sequential hits, the incidence curve would require a latent period to rise (as seen in the 0.16 Gy group). The fact that 30.4% of high-dose tumors manifested almost immediately suggests that the “one-off” physical insult of radiotherapy functioned as a simultaneous multi-hit event, effectively bypassing the temporal constraints of traditional carcinogenesis. Third, regarding the implication of “punctuated equilibrium”: Stephens et al. posit that cancer evolution can occur through rapid transformation rather than gradual change. The “Diagnostic Trap” observed in RI-MPM exemplifies this mechanism. In standard carcinogenesis, the gradual accumulation of mutations allows time for morphological adaptation, such as Type 3 EMT. However, chromothripsis enables the tumor to acquire aggressive compound drivers (e.g., simultaneous loss of *CDKN2A* and *NF2* and/or *BAP1*) instantaneously. Because this molecular evolution outpaces biological time, the tumor exhibits a “morphological lag”: it harbors the lethal kinetics of a sarcomatoid tumor while retaining the deceptively innocent architecture of an epithelioid one. Critically, this mechanism appears dose-threshold-dependent. The lower-dose group (0.16 Gy) did not exhibit the “Step-Jump” signature, showing instead a selection bias pattern (high early exclusion of susceptible individuals) followed by gradual kinetics. This suggests that the energy required to trigger “Event Zero” exceeds the levels used in intermediate protocols. Only doses crossing a physical threshold—potentially ≥45 Gy in human thoracic irradiation—appear sufficient to shatter the genome and trigger this instantaneous “Double-Brake” failure.

### 4.6. Strengths and Limitations

The primary limitation underlying the proposed risk-stratified framework is the small sample size (*n* = 20), which inherently restricts statistical power. However, this limitation is the deliberate result of our stringent inclusion criteria designed to utilize IPD analysis effectively. Unlike aggregate data from large databases, our IPD approach prioritized the strict exclusion of asbestos exposure to ensure the isolation of a pure radiotherapy-specific phenotype. Recognizing that reliance on historical case reports inherently limits the verification of asbestos exposure, we mitigated this risk by cross-referencing clinical findings against exposure history whenever possible. For instance, our screening process identified and excluded a candidate case that claimed no exposure history but was found to harbor pleural plaques, underscoring the rigor of our selection despite data limitations.

We explicitly acknowledge publication bias—where dramatic or rapidly progressing cases are preferentially reported—as a systematic error. While we cannot fully quantify this bias without access to unpublished negative cases, we mitigated its impact through comprehensive searching and transparent inclusion criteria. Furthermore, as this dataset is derived exclusively from a synthesis of historical case reports, physical access to biological specimens for molecular validation was methodologically precluded.

Consequently, while this study establishes a physical framework to characterize the ‘Diagnostic Trap’, it remains primarily hypothesis-generating regarding the underlying molecular mechanisms. Given that the relationship between radiotherapy dose and molecular phenotypes remains unexplored in RI-MPM, we developed the ‘Single- to Double-Brake’ model as a plausible theoretical framework that aligns with the physical basis identified in our cross-species analysis. Regarding this cross-species comparison, absolute dose values differ between human and canine studies, consistent with their respective exposure protocols. Beagles in the CSU study received 60Co gamma-radiation exposure at various doses according to the CSU experimental design [37].

Thus, the canine model (*n* = 829) provides cross-species validation for the dose-dependent radiation effects identified in our clinical cohort. We acknowledge that this canine validation addresses the dose-dependent patterns of tumor latency rather than the ‘Diagnostic Trap’ itself, which remains a hypothesis specific to human RI-MPM requiring prospective molecular validation.

Therefore, our proposal to use radiotherapy history as a surrogate marker should be interpreted as a pragmatic strategy for risk stratification rather than established high-level evidence. Nevertheless, while prospective Randomized Controlled Trials (RCTs) are not feasible for this ultra-rare etiology, international case registries represent a realistic validation pathway. In the interim, this framework provides a conceptual compass for clinicians. It offers a physical framework that fills a critical knowledge gap where no other guidance currently exists, justifying molecular scrutiny regarding the “Diagnostic Trap” in high-dose cases.

## 5. Conclusions

This study elucidates the kinetic distinctness of high-dose radiation carcinogenesis. The large-scale canine model (*n* = 829)—encompassing diverse radiation-induced malignancies—validates the universality of “Event Zero” kinetics, where high-dose exposure triggers immediate tumor onset. Our human IPD analysis (*n* = 20) translates this physical law into the specific clinical context of RI-MPM, identifying the “Diagnostic Trap”: a scenario where the instantaneous molecular aggression driven by high-dose radiotherapy is paradoxically masked by an indolent epithelioid morphology.

To explain this decoupling, we propose the ‘Double-Brake Failure’ model. This framework offers a mechanistic rationale for why high-dose physical insults can bypass gradual morphological dedifferentiation, leading to rapid clinical progression inconsistent with histological appearance. While molecular validation is the necessary next step, the physical evidence of instantaneous onset identified here fundamentally challenges the current reliance on morphology alone.

Consequently, we advocate for a paradigm shift in risk stratification: radiotherapy dose history should serve as a critical “red flag” independent of histological grade. For patients with a history of high-dose exposure, bland epithelioid histology should not be interpreted as a sign of indolence. Instead, the physics of their exposure mandates high-alert surveillance and justifies molecular profiling, filling a critical guidance gap where standard pathological assessment is fundamentally insufficient.

## Figures and Tables

**Table 1:** Malignant Pleural Mesothelioma in Patients with a History of Radiotherapy.

Case	Type of the First Tumor	RT Dose (Gy)	Sex	Age at Radiotherapy	Age at Diagnosis	Latent Period (Years)	Chemo-Therapy	Histology	Reference
1	Hodgkin’s	Unknown	M	29	34	5	Unknown	Sarc	[10]
2	Hodgkin’s	Unknown	M	27	34	7	Unknown	Sarc	[11]
3 §	Breast cancer	46	F	30	40	10	AA	Epi	[12]
4 §	Seminoma	30	M	33	57	24	Unknown	Epi	[13]
5	Wilms’ tumor	Unknown	M	3	44	41	Unknown	Epi	[14]
6	Wilms’ tumor	34 ^b^	M	6	22	16	AA	Not reported	[14]
7	Wilms’ tumor	15 ^a^	M	2	16	14	Chemo	Epi	[15]
8 †	Breast cancer	Unknown	F	34	64	30	Unknown	Epi	[16]
9 §	Hodgkin’s	36	F	4	24	20	Unknown	Epi	[17]
10	Hodgkin’s	40 ^b^	F	13	22	9	Unknown	Not reported	[18]
11	Breast cancer	50 ^c^	F	65	75	10	None	Epi	[19]
12	Breast cancer	45 ^b^	F	37	72	35	None	Epi	[19]
13	Hodgkin’s	Unknown	M	28	49	21	None	Epi	[20]
14	Hodgkin’s	40 ^b^	F	21	43	22	None	Biph	[20]
15	Hodgkin’s	42 ^b^	M	20	31	11	AA + AC	Epi	[20]
16	Breast cancer	Unknown	F	49	78	29	None	Epi	[20]
17	Hodgkin’s	35 ^b^	M	32	46	14	AA	Epi	[21]
18	Hodgkin’s	35 ^b^	M	7	32	25	AA	Epi	[21]
19	Hodgkin’s	38 ^b^	M	7	18	11	None	Epi	[22]
20	Sertoli-Leydig cell tumor	36.5 ^b^	F	11	20	9	AA	Epi	[22]
21	Hodgkin’s	36 ^b^	M	40	64	24	Unknown	Not reported	[23]
22	Hodgkin’s	Unknown	F	18	30	12	Unknown	Epi	[24]
23	Lung cancer	60 ^c^	F	49	66	17	Unknown	Epi	[25]
24	Breast cancer	Unknown	F	50	60	10	Chemo	Epi	[26]
25	Diffuse large B-cell lymphoma	Unknown	F	29	45	16	Unknown	Epi	[27]
26	Hodgkin’s	Unknown	F	22	31	9	Unknown	Epi	[27]
27	Hodgkin’s	Unknown	F	22	54	32	Unknown	Epi	[27]
28	Hodgkin’s	50 ^c^	F	25	50	25	AA	Epi	[28]
29	Breast cancer	50 ^c^	F	59	75	16	None	Not reported	[29]
30	Breast cancer	60 ^c^	F	66	68	2	None	Not reported	[29]
31	Breast cancer	60 ^c^	F	44	54	10	None	Not reported	[29]
32	Hodgkin’s	40 ^b^	F	33.2	66.2	33.0	Chemo	Biph	[30]
33	Hodgkin’s	40 ^b^	F	31.8	65.9	34.1	Chemo	Epi	[30]
34	Hodgkin’s	40 ^b^	F	22.1	49.8	27.7	Chemo	Epi	[30]
35 §	Hodgkin’s	40	F	15.4	49.3	33.9	None	Epi	[30]
36 §	Hodgkin’s	40	F	21.8	40	18.2	Chemo	Epi	[30]
37 ‖	Hodgkin’s	35	M	33.1	43.6	10.5	Chemo	Epi	[30]
38 §	leukemia	Unknown	M	5	34	29	Unknown	Epi	[31]

Hodgkin’s: Hodgkin’s lymphoma. § Excluded from the analysis to avoid confounding bias from intervening treatments for a tertiary cancer. † Dose entered as ‘unknown’ per original report; the 45 Gy value from a review by Sekine et al. was not used. ‖ Excluded from the analysis due to uncertainty that the tumor arose within the radiation field. ^a^ Low-dose group (<20 Gy). ^b^ Intermediate-dose group (20–45 Gy). ^c^ High-dose group (>45 Gy). Abbreviations: AA, alkylating agent; AC, anthracycline; Chemo, chemotherapy (unspecified); Epi, epithelioid; Biph, biphasic, Sarc, sarcomatoid. Note: Background shading is used to distinguish between different references for better readability.

**Table 2:** Cumulative Incidence of Terminal Disease Requiring Euthanasia by Time Point and Radiation Dose (CSU Beagle Study).

Time Point (Years)	0 Gy	0.16 Gy	≥0.74 Gy
0.5	1.4% (2)	1.7% (3)	30.4% (155)
1	2.1% (3)	1.7% (3)	31.8% (162)
2	18.1% (26)	2.3% (4)	36.9% (188)
3	26.4% (38)	2.3% (4)	37.3% (190)
5	28.5% (41)	2.3% (4)	38.4% (196)
7	32.6% (47)	2.9% (5)	40.6% (207)
10	42.4% (61)	17.7% (31)	48.6% (248)
12	62.5% (90)	36.0% (63)	62.9% (321)
15	86.8% (125)	80.6% (141)	90.0% (459)
20	100.0% (144)	100.0% (175)	100.0% (510)

Values are shown as percentage (absolute number of cases). The total number of cases is *n* = 829.

## Data Availability

Publicly available datasets were analyzed in this study. The human Individual Patient Data (IPD) used to characterize the “Diagnostic Trap” in RI-MPM were synthesized from previously published literature and are summarized in Table 1. The canine datasets, which provide the quantitative basis for dose-dependent kinetic patterns underlying the Diagnostic Trap, are publicly available via the Northwestern University Radiation Archive (NURA) at https://sites.northwestern.edu/nura/data/colorado-state-university/ (accessed on 6 January 2026). These records originate from the Colorado State University (CSU) Beagle Study, funded by the National Cancer Institute and the Department of Energy. Additionally, TCGA mesothelioma genomic data can be accessed at the cBioPortal for Cancer Genomics (https://www.cbioportal.org/ (accessed on 6 January 2026)), and the statistical analysis code generated for this study is available in the Supplementary Materials (Files S1 and S2).

## Supplementary Materials

The following supporting information can be downloaded at https://www.mdpi.com/article/10.3390/cancers18020221/s1, Figure S1: Distribution of Latent Period by Age at Radiotherapy; Figure S2: Bootstrap distributions of Spearman’s correlation coefficients; Table S1: Methodological quality assessment; File S1: R statistical code for human RI-MPM analysis; File S2: R statistical code for canine cumulative incidence analysis.
