# Supplementary material for: The Diagnostic Trap in Radiation-Induced Mesothelioma: Kinetic-Morphological Decoupling Masks Molecular Aggression
Source: Cancers (Basel). 2026 Jan 9;18(2):221. doi: 10.3390/cancers18020221 (PMC12839075; doi:10.3390/cancers18020221)
Supplement: Supplementary file 1 [file cancers-18-00221-s001.zip › Cancers_Supplemental.pdf]

Supplementary Table 1. Methodological quality assessment using the Murad et al. framework.

Checklist Item	Corresponding Section in Manuscript & Specific Content
**Selection**	
1. Does the patient(s) represent(s) the whole experience of the investigator (centre) or is the selection method unclear to the extent that other patients with similar presentation may not have been reported?	**Data Source and Case Identification:**<br>This study is a systematic search and review, not a single-center case series reporting on an investigator's personal experience. To minimize selection bias, we systematically identified published cases that meet explicit criteria, using a reproducible search of PubMed, backward citation, and a foundational study by Sekine et al. This process ensures a transparent and comprehensive case collection.
**Ascertainment**	
2. Was the exposure adequately ascertained?	**Table 1 / Data Source and Case Identification:**<br>Yes, within the inherent limitations of a literature review based on historical case reports. The primary exposure, radiotherapy dose, was ascertained from the original publications and is listed in Table 1. We acknowledge that dose reporting standards and techniques have varied over time. To ensure the best possible data quality for our analysis, cases with missing dose data were rigorously excluded. Furthermore, the retrieved doses were stratified using established oncological guidelines to allow for systematic comparison. For a review of this rare entity, this represents the most systematic and adequate ascertainment feasible.
3. Was the outcome adequately ascertained?	**Table 1:**<br>Yes, with the understanding that the outcome is derived from data in historical reports. The primary outcome, latent period, was consistently calculated as the difference between the age at diagnosis and the age at radiotherapy, with both data points extracted from the source publications (Table 1). We acknowledge that the precise definition of 'age at diagnosis' may vary between reports. However, by applying a uniform calculation to the explicitly reported data, we ensured a systematic and reproducible ascertainment of the outcome. This represents the most rigorous approach feasible for this literature-based study.
**Causality**	
4. Were other alternative causes that may explain the observation ruled out?	**Data Source and Case Identification:**<br>Yes, to the extent feasible within a literature-based study. The primary alternative cause for MPM, asbestos exposure, was systematically ruled out as a core inclusion criterion. The significant confounding effect of chemotherapy was also specifically analyzed. We acknowledge that other factors, such as an underlying genetic predisposition (e.g., BAP1 syndrome), could also contribute to pathogenesis. However, this level of genetic information is not available in the historical case reports reviewed. Therefore, our study controlled for the most critical and assessable alternative causes based on the available data.
5. Was there a challenge/rechallenge phenomenon?	**Not Applicable:**<br>This item is primarily relevant to adverse drug events. It is not applicable to radiation-induced malignancy, which involves a single historical exposure event followed by a long latency period.
6. Was there a dose-response effect?	**Overview: Dose-Stratification Reveals Opposing Trends/ Discussion:**<br>Yes, a complex, non-linear dose-response relationship was the central focus of this study. Our analysis revealed opposing, dose-dependent trends in the relationship between age and latent period across different dose strata. This observation led to the formulation of our 'Dose-Dependent Pathogenic Hypothesis', which was specifically proposed to explain how different radiation doses could result in different pathogenic mechanisms and clinical timelines.
7. Was follow-up long enough for outcomes to occur?	**Table 1:**<br>Yes. The study inherently includes cases with long follow-up, as RI-MPM is a disease with significant latency. The latent periods documented in Table 1 range from 2 to 41 years, which is adequate for this outcome to occur.
**Reporting**	
8. Is the case(s) described with sufficient details to allow other investigators to replicate the research or to allow practitioners make inferences related to their own practice?	**Table 1 / Materials and Methods:**<br>The response to this question has two parts:<br>**For Replicating the Research:** Yes. The manuscript provides all necessary details to replicate our analysis. We provide a full list of the cases reviewed with their original citations (Table 1), the complete dataset used for analysis (Table 1), and a detailed description of our literature search and statistical methods.<br>**For Practitioner Inferences:** Limited. This study proposes a research hypothesis based on historical case reports, not clinical guidance. These reports lack uniform data, especially on critical prognostic factors like histological subtype, precluding direct application to patient care. The primary value for practitioners is in understanding a framework that may inform future therapeutic research.

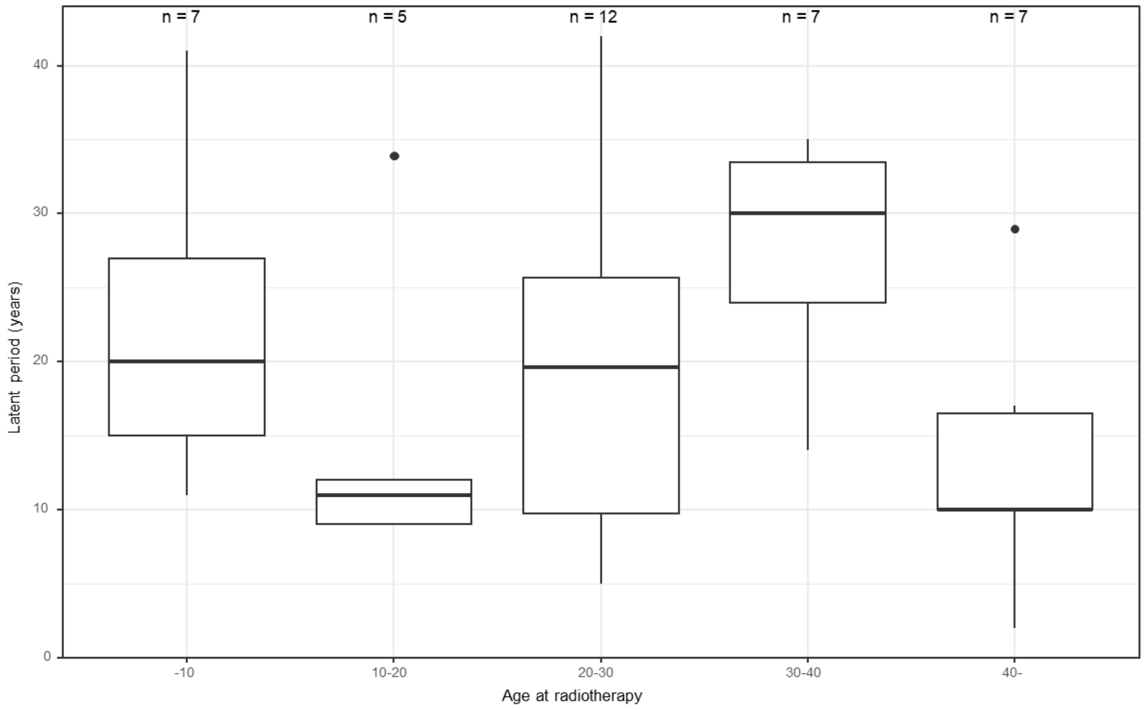

Supplementary Figure 1. Distribution of Latent Period by Age at Radiotherapy.
A boxplot illustrating the distribution of the latent period for all 38 identified cases, stratified into five age-at-radiotherapy groups: <10, 10–20, 20–30, 30–40, and ≥40 years. The central line in each box indicates the median, with the box representing the interquartile range (IQR). Whiskers extend to 1.5 times the IQR, and outliers are shown as dots. The plot demonstrates a complex, non-linear relationship, which provided the rationale for the dose-stratified analysis performed in the main manuscript by demonstrating that a single linear regression model is statistically inappropriate for the pooled cohort.

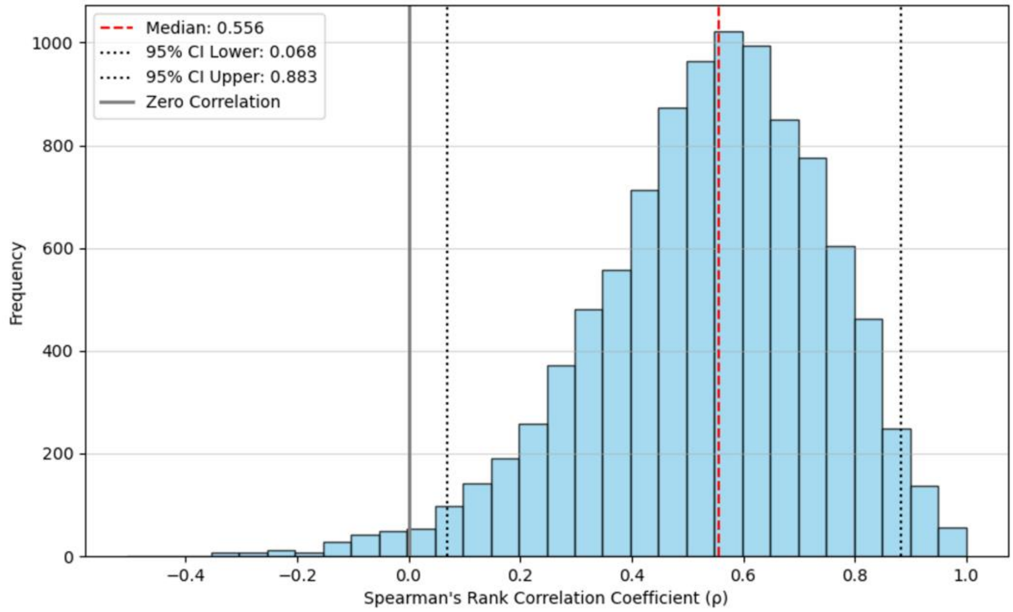

(A) Intermediate-dose group

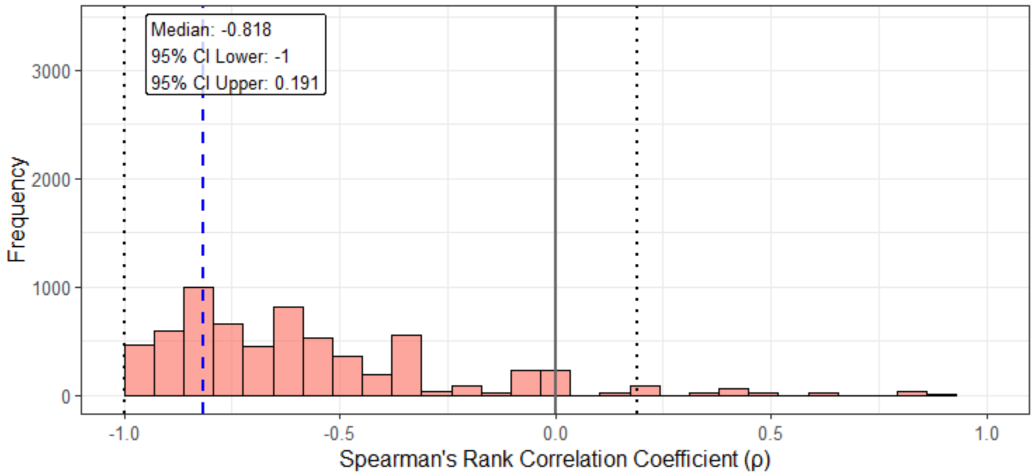

(B) High-dose group

Supplementary Figure 2. Bootstrap distributions of Spearman's correlation coefficients, demonstrating the stability of the observed trends.

In each panel, dashed lines indicate the median and dotted lines indicate the 95% confidence interval. (A) In the intermediate-dose group, the 95% confidence interval of the Spearman's coefficient [0.068, 0.883] does not include zero. Although wide, this interval supports a positive correlation within this cohort. (B) In the high-dose group, the 95% confidence interval [-1.000, 0.191] includes zero, confirming the lack of statistical significance for the negative trend.
